# Reducing social isolation during the COVID-19 pandemic: Assessing the contribution of courtesy phone calls by volunteers

**DOI:** 10.1371/journal.pone.0266328

**Published:** 2022-05-04

**Authors:** Louise Normandin, Caroline Wong, Vincent Dumez, Kathy Malas, Alexandre Grégoire, Julie Grégoire, Lise Pettigrew, Nicolas Allanot, Cécile Vialaron, Sabrina Anissa El Mansali, Christine Nguyen, Fabrice Brunet, Marie-Pascale Pomey

**Affiliations:** 1 Innovation Axis, Research Center of The Centre Hospitalier de l’Université de Montréal, (CHUM), Montréal, Québec, Canada; 2 Center of Excellence on Patient Partnership and The Public, Montréal, Québec, Canada; 3 Faculty of Medicine, Université de Montreal, Montréal, Québec, Canada; 4 General Directorate, Centre hospitalier de l’Université de Montréal, Montréal, Québec, Canada; 5 Education and Academy Directorate, Centre Hospitalier de l’Université de Montréal, Montréal, Québec, Canada; 6 Volunteer, Recreation and Leisure Department, Centre Hospitalier de l’Université de Montréal, Montréal, Québec, Canada; 7 Department of Health Management, Evaluation, and Policy, School of Public Health, Université de Montréal, Montréal, Québec, Canada; 8 Research Chair in Evaluation of State-of-the-Art Technologies and Methods, Montréal, Québec, Canada; University of Auckland, NEW ZEALAND

## Abstract

**Context:**

During the COVID-19 pandemic, restrictions were imposed on visits in hospitals in the province of Quebec, Canada in an effort to reduce the risk of viral exposure by minimizing face-to-face contact in order to protect patients, visitors and staff. These measures led to social isolation for patients. In order to reduce this isolation, CHUM (the Centre hospitalier de l’Université de Montréal, a teaching hospital) shifted from in-person visits to courtesy telephone calls delivered by volunteers from CHUM’s Volunteers, Recreation and Leisure Department.

**Objectives:**

To study: (1) the contribution made by these calls to reducing isolation and their limitations, (2) how the calls can be improved, and (3) whether they should be maintained, based on the views of patients and volunteers.

**Methodology:**

This study examined two populations. The first one consisted of 189 adult patients hospitalized at CHUM who received a courtesy phone call from a volunteer and the second one consisted of the 25 CHUM volunteers who made these calls. Quantitative data were collected from patients and volunteers through questionnaires and a Smartsheet. The patient questionnaire evaluated isolation, the courtesy phone calls, the relationship of trust with the volunteer and sociodemographic questions. The volunteer questionnaire evaluated the appropriateness of the technology for the intervention, the support and training received, the impacts of the courtesy phone call on both the patients and the volunteers, an experience report and sociodemographic information. In addition, a focus group was held with 7 volunteers. Then the verbatim were transcribed and analyzed using QDA miner software.

**Results:**

From April 27, 2020 to September 5, 2020 more than 11,800 calls were made, mainly concerning hospitalization conditions or home follow-ups (n = 83), and relationships with relatives, friends, and family (n = 79). For 73.6% of hospitalized patients, the courtesy calls from volunteers were a good response to their needs, and 72% of volunteers agreed. 64.5% of patients felt less isolated and 40% of volunteers felt useful.

**Conclusion:**

Our data suggest that patients felt less isolated during their hospitalization because of the courtesy calls made by the volunteers, that smartphones could also be used for video calls and, finally, that maintaining this type of service seems as relevant after as during a pandemic to provide social interactions to people isolated for medical reasons.

## Introduction

Faced with the unprecedented health situation related to the SARS-CoV-2 pandemic [1], the government of the province of Quebec, Canada declared a health emergency on March 13, 2020 (section 118 of the *Public Health Act* (chapter S-2.2) [2]. With the implementation of this exceptional measure and to protect the health of the population (section 123), the government ordered the prohibition of visits to hospitals and long-term care centers (CHSLDs) [2] in order to limit the spread of COVID-19 in these institutions. However, these measures can lead to the isolation of individuals, which has adverse effects on mental health [3]. Moreover, previous studies have shown that feelings of loneliness have negative consequences on both mental and physical health and that they can significantly increase the risk of death, particularly among older men [4]. In this context, and to reduce social isolation and feelings of loneliness among hospitalized patients, while limiting the risk of contamination, the Centre hospitalier de l’Université de Montréal (CHUM), facilitated by its School of Artificial Intelligence in Healthcare [5] and all its departments, including the Centre of Excellence on Patient and Public Partnership, implemented several social and technological innovations, one of which consists in organizing courtesy phone calls from volunteers at CHUM’s Volunteers, Recreation and Leisure Department. This Department has 975 volunteers, who are all trained prior to their volunteering in patient experience and interaction and are supported continuously in the organization by five permanent employees. It intervenes at the clinical level by welcoming, orienting and visiting patients, visitors and attendants as well as by participating in facilitation and leisure activities or by helping with vaccination campaigns, public events, administrative tasks for certain sectors, etc.

To implement these courtesy phone calls to all hospitalized patients, CHUM’s Volunteer Recreation and Leisure Department sent an e-mail to all 1,000 volunteers inviting them to participate in this new form of volunteering. Normally the hospital’s volunteers would provide listening and support at bedside. A total of 70 volunteers expressed an interest in getting involved. The Volunteer Activities and Recreation Department then developed a training program in the form of a 3-hour webinar that would to enable volunteers to: (1) understand the context of the pandemic and its impact on hospitalized patients, their loved ones, caregivers and the community, (2) understand the objectives of the courtesy phone calls and their role; (3) learn how to use the Smartsheet platform [6] (accessing lists of patients, calling for follow-up), and (4) participate in a study. In addition, webinars following the initial training allow interested volunteers to share their experiences during courtesy calls with other volunteers as a way to improve their interventions.

For the volunteers, the courtesy phone calls consisted of receiving a list of hospitalized patients each day and calling them according to their availability. When the patient could be reached, the volunteer would introduce himself or herself, inquire about the patient’s condition and enter into a general discussion based on the needs. Following each courtesy phone call, the volunteer would document the length of the call and the topics discussed. Although various initiatives aimed at reducing social isolation, such as telephone contact [7, 8] and video telephony using robots [9], were implemented in health systems during the COVID-19 pandemic [10], to our knowledge, few initiatives have been implemented in hospital settings [8, 11, 12].

Therefore, the objective of this study is to evaluate the perceived contribution of these courtesy phone calls to reducing the effects of social isolation among hospitalized patients in the context of the first wave of COVID-19 as well as the volunteers’ perceptions of the contribution made by these calls. More specifically, this study investigates patients’ and volunteers’ points of view on: (1) the perceived contribution and limitations of these calls in terms of patient isolation, (2) how they could be improved, and (3) whether they should continue in the future.

## Materials and methods

### Study populations

This study examined two populations. The first one consisted of adult patients hospitalized at CHUM who received a courtesy phone call from a volunteer in the period from May 28 to September 5, 2020 and the second one consisted of the CHUM volunteers who made these calls.

### Patient recruitment

The criteria for selecting patients for the study were: patients whose courtesy call with a volunteer lasted at least 5 minutes. At the end of the call, the volunteer asked the patient whether he or she would agree to be called by the research team to assess his or her perceptions of the contribution made by the call. If the person agreed, a member of the research team would contact the person within 24 hours of the call to present the research project and obtain oral consent for participation in the study. If the patient agreed to participate, the questionnaire was completed immediately by phone with a member of a research team. Up to three follow-up calls were made.

### Volunteer recruitment

All the volunteers who participated in the courtesy phone calls were approached to participate in the study by managers in the Volunteers, Recreation and Leisure Department. No exclusion criteria were applied.

### Questionnaires

A review of the literature did not identify any questionnaires that met the specific objectives of our study, so we drew inspiration from two questionnaires: one dealing with psychological distress [13] and the other with the partnership relationship with stakeholders [14]. The goal was to construct two questionnaires that would take into consideration the health crisis context. The questionnaires were developed with input from three CHUM patient partners for the patient questionnaire and from two volunteers for the volunteer questionnaire. The questionnaires were pre-tested on samples of patients (n = 10) and volunteers (n = 4) to ensure that the questions would be understood and to estimate the time required to answer them.

The patient questionnaire comprises eight questions that evaluate isolation (n = 1) [13], the courtesy calls (n = 2) and the relationship of trust with the volunteer (n = 1) [14]. It also includes sociodemographic questions (n = 4). At the end of the questionnaire, the patient can add a comment. The questionnaire takes about 5 minutes to complete (S1 and S2 Files).

The volunteer questionnaire evaluates 6 dimensions and comprises 13 questions. It takes approximately 5 minutes to complete and documents, for each call, the appropriateness of the technology for the intervention (n = 3), the support and training received (n = 3), the impacts of the courtesy phone call on both the patients and the volunteers (n = 3), an experience report (n = 2) and sociodemographic information (n = 2). In addition, there is a space for comments at the end of the questionnaire (S3 and S4 Files).

Responses to each question in both questionnaires are given on a 5-point Likert scale (completely disagree / somewhat disagree / neutral / somewhat agree / completely agree). Participants could also answer: I don’t want to answer / I don’t know / Does not apply.

### Focus group of volunteers

At the end of the first wave of the pandemic, the volunteers were invited to participate in a focus group to assess their reasons for participating in these calls, the difference between making phone calls versus face-to-face visits, and how the relationship of trust between volunteers and patients was established during the calls (S5 File). The focus group consisted of seven volunteers and was facilitated by two people with experience in qualitative research (MPP and LN). It lasted 60 minutes and was video recorded.

### Data analysis

For the survey data, descriptive statistics were calculated using REDCap (Research Electronic Data Capture), an application for building and managing the online surveys and databases used to administer questionnaires, organize data collection and analyze data [15].

The focus group’s discussions were transcribed verbatim by LN, reviewed by MPP, and analyzed using QDA miner software [16]. MPP and LN identified, independently of each other, quotes that could illustrate the answers to the questions. They then met to discuss their respective choices and determine which quotes best illustrated the participants’ ideas. The same process was followed for the comments provided on the questionnaires. The consolidated criteria for reporting (COREQ) checklist [17] was used to report the methodology used with the focus group.

### Ethics approval

This study received ethical approval from the Research Ethics Committee (20.040) of the Centre de recherche du Centre hospitalier de l’Université de Montréal (CRCHUM).

## Results

### Characteristics of the participants

In the study period from May 28 to September 5, 2020, 663 patients received phone calls from volunteers. Of these, 126 (19%) had left the hospital at the time of data collection, 176 (27%) did not respond, 37 (5%) indicated that they did not receive a courtesy phone call, and 135 (20%) refused to respond due to health or hearing problems or lack of interest and time. Therefore, a total of 189 patients agreed to answer the questionnaire, for a participation rate of 28.5%.

The patients ranged in age from 19 to 100 years, with a mean age of 66 years. 9% of patients (n = 16) were hospitalized for COVID-19, while the rest were hospitalized for other conditions. The patients hospitalized for COVID-19 were 57–96 years of age, with an average age of 78 years.

A total of 69 volunteers expressed an interest in participating in the courtesy phone calls, 57 of whom made calls and filled out the Smartsheet after each call. 25 responded to the questionnaire (participation rate = 44%). 76% (n = 19) of the respondents were women.

### Courtesy phone calls delivered by volunteers

#### General results

Between April 27, 2020 and September 5, 2020, more than 11,800 calls were made, for a total of 39,730 minutes of conversation (equal to 662 hours) and an average of 9 minutes per call.

The 6 topics most frequently discussed during the courtesy phone calls were the conditions of their hospitalization or follow-up at home (n = 83), relationships with family and friends (n = 79), illness and symptoms (n = 49), feelings of isolation (n = 24), emotions (n = 13), and distractions in general, such as news, movies, and television (n = 12).

#### Patients’ answers

**Fig 1:**
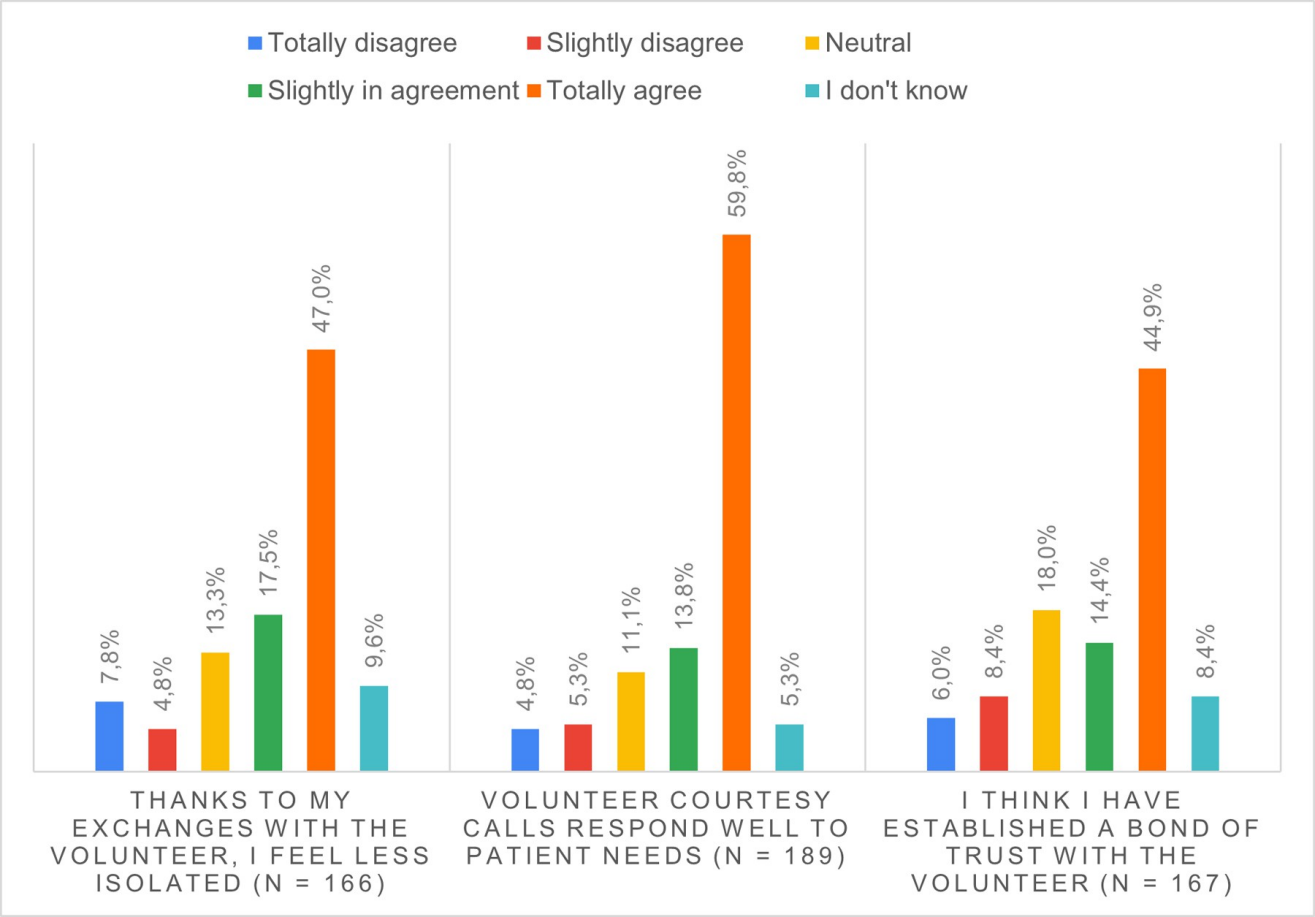
Patients’ perceptions of the volunteer courtesy phone calls.

For 73.6% of the hospitalized patients, the courtesy phone calls from volunteers were an appropriate response to their needs. 64.5% felt less isolated and 59.3% created a bond of trust with the volunteer (Fig 1).

The majority of patients (69.7%) had no problem with receiving a courtesy phone call from a volunteer. However, a few patients highlighted certain disadvantages, such as a lack of interest in these calls (5%, n = 9), fear over the confidentiality of the information exchanged (1%, n = 2), too many calls received (3%, n = 5), not enough calls received (2%, n = 4), the call being too short (2%, n = 3), the inappropriate timing of the call (3%, n = 5), different volunteers on each call (3%, n = 5), the volunteer’s difficulty in understanding the patient (1%, n = 1), and a preference for speaking with a professional (1%, n = 2).

Three types of ideas emerged from the comments made by patients at the end of the questionnaire: (1) the contribution made by these calls, (2) the limitations of this type of social support, and (3) the nature of these calls (Table 1).

**Table 1:** Comments made by patients on the courtesy phone calls*.

Contribution made	Limitations of the calls	Nature of the calls
“The volunteers’ calls are sweet and courteous.” (ID 623) “I would like to continue receiving calls from volunteers.” (ID 439) “The volunteers are great.” (ID 660)	“[…] I have family and friends calling me, but I find the concept interesting.” (ID 597) “The calls from volunteers are a good initiative, but personally, I have enough support and don’t need it.” (ID 228) “I don’t need volunteers’ calls, I already have enough support. But I understand that it’s useful for people who have few relatives.” (ID 374)	“The conversation was boring.” (ID 71) “I need to talk to professionals more than volunteers.” (ID 169) “I prefer to talk to a professional, like a psychologist.” (ID 478) “[…] our discussion was particularly brief.” (ID 92) “It is a different volunteer with each call, so you can’t create much of a bond of trust.” (ID 333)

*The quotes have been translated from French.

### Volunteers’ answers

**Fig 2:**
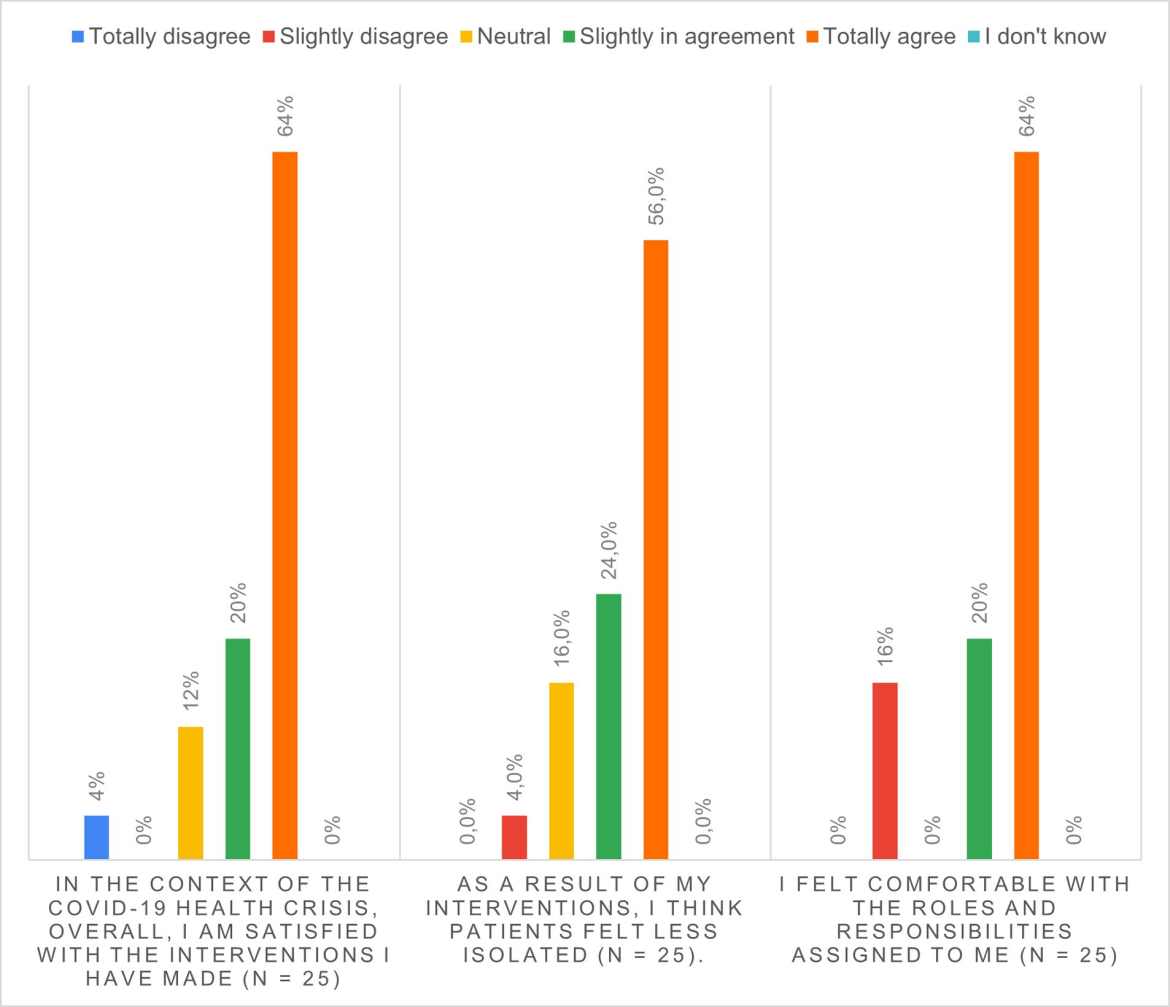
Volunteers’ perceptions of the courtesy phone calls they made to patients.

The results from the questionnaires show that 84% of the volunteers were satisfied with their interventions with patients. For 80% of them, they believe that the patients felt less isolated as a result of their interventions. 84% felt comfortable with the roles and responsibilities assigned to them (Fig 2).

**Fig 3:**
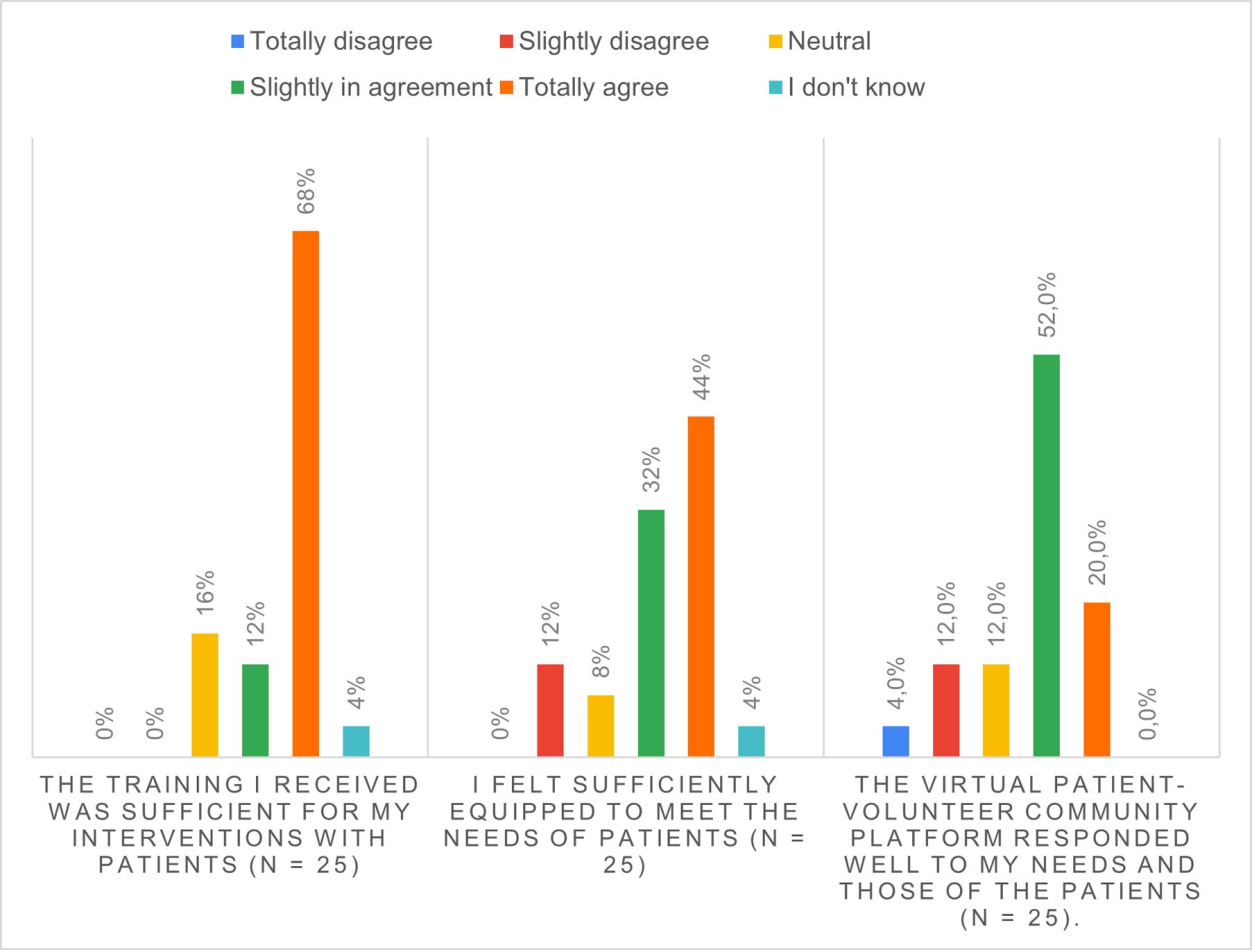
Volunteers’ perceptions of the conditions under which the calls were made.

80% felt that the training they received was sufficient for interacting with patients, and 76% felt they were sufficiently well equipped to respond to patient needs. 72% of the volunteers found that the web platform met their needs (Fig 3).

For the volunteers, their involvement had a positive impact in terms of making them feel useful (40%, n = 10), giving to others what they themselves had received (24%, n = 6), developing new skills (12%, n = 3), feeling valued (8%, n = 2) and feeling less isolated (8%, n = 2).

However, among those who identified negative impacts, 20% did not feel up to the task or, more marginally, 4% of the volunteers did not feel sufficiently supported in their interventions, 4% felt that they were insufficiently trained, 4% felt that they were under too many constraints, and 4% feared that they did not fully understand their contribution.

Lastly, three main themes emerged in the focus group: (1) the contribution made by these calls; (2) the difficulties encountered; and (3) the improvements that could be made to the intervention.

In terms of the contribution made by these calls, one of the volunteers said: "It reassures patients to know that they are not alone. The response from patients, even if they don’t talk to me for long, is always good. They usually appreciate it a lot." (Volunteer 1). The volunteers also enjoyed making the courtesy calls. One volunteer said, "I’ve had calls that were beyond my expectations in terms of going to the essence of what two humans can say to each other. At one point I chatted with a fellow for an hour. It was delightful." (Volunteer 5). Another said, "It gives me great satisfaction. […] 90% of the patients are happy. Some of them want to tell us their life story, but after 25 minutes we have to move on to another call." (Volunteer 2). Moreover, it seems that these calls lead to specific behaviours:

“On the phone, men react differently than they do in person. They are more talkative. Face to face, women are more talkative than men." (Volunteer 3)

“Women are easier to approach than men, but this is not always the case. Some evenings, there are as many men as women who will talk to me." (Volunteer 1)

They also emphasized the difficulties encountered making calls compared to face-to-face exchanges. This was also reflected in the comments provided in the questionnaires (Table 2).

**Table 2:** Feedback from volunteers on the limits of courtesy phone calls*.

Comments in questionnaires	Focus group
“The calls were appreciated by most patients, but nothing can replace physical contact for getting people to feel less isolated.” (Volunteer 9) “Volunteer work over the phone is not as pleasant as in person. For many older patients, it isn’t easy.” (Volunteer 21) “The biggest challenge is not being able to use body language to build trust with the patient.” (Volunteer 38) “Many patients were on the defensive, and feared they would be tricked. Listening on the phone and a face-to-face visit is as different as day and night. It isn’t easy for the patient or the caller.” (Volunteer 8)	“At the beginning it’s difficult. You look for an angle for getting the patients talking. I found my angle: I use humour.” (Volunteer 2) “What I find difficult on the phone, it’s that we’re not there, you can’t see if people are asleep. […]. I’m not comfortable on the phone because you can’t see the person. A face-to-face approach is easier.” (Volunteer 1) “We don’t see the non-verbal clues, and we invite ourselves into the person’s room (we don’t know if a nurse is present) without seeing the person’s condition.” (Volunteer 3) “The discussions are shorter than face-to-face conversations.” (Volunteer 4) “I find calling patients every day a burden. [. . .] We are cut off from the person’s humanity.” (Volunteer 5)

*The quotes have been translated from French.

In terms of ways to improve the intervention, several volunteers mentioned the importance of having the same volunteer call a patient throughout his or her stay in order to build a relationship of trust. This makes it possible to: "In some cases, […] to continue the conversation we had last time. (…) It’s a warmer call, because it’s someone who recognizes me" (Volunteer 1). Such a mechanism brings us closer to a face-to-face visit:

"When a patient had been hospitalized for a long time, I would visit him or her every time I went to CHUM. There was a bond of trust. The patient was happy to see me arrive. Some didn’t want to see volunteers, but if they knew it was me, they’d tell me to come in. It’s true that we’re not there to make friends, but as volunteers, sometimes it helps." (Volunteer 1)

Even if it is not a friendship, being able to talk with patients you already know can avoid the discomfort of the first call:

"There would be a level of trust that would take hold. The discussion would no longer be at the same level on the second call, especially with patients who are hospitalized for a long time. On the phone, we don’t know the person, and they don’t know us, either. There is some initial discomfort. […] One person asked me if I was going to call her back.” (Volunteer 4).

"At the beginning of the pandemic, I was volunteering at a community center for seniors. Calls are made once a week. I had the same list every week. I enjoyed the experience. I could see that when we talked to people a little bit more, they were more natural and more trusting. I go out of my way to see people I know. I feel that the presence of a volunteer, like me, was comforting to the patient because it provides continuity. Patients like to talk to us again." (Volunteer 5)

However, this perception was not shared by one of the volunteers:

“We drifted away from the goal of making courtesy calls. I’m not in a helping or active listening relationship with someone who I’m going to follow for weeks. I don’t want to have a special relationship and find myself dealing with people’s problems. I don’t want to be their friend. I will avoid getting too involved with someone. It would get too personal.” (Volunteer 2)

Lastly, on the issue of whether these courtesy calls should be maintained, the volunteers expressed an interest in maintaining them throughout the COVID-19 crisis, and also suggested going beyond that, but as a complement to on-site visits for some volunteers who cannot travel or to be in contact with patients who are feeling isolated. The younger volunteers also wanted these calls to continue, as they give them greater flexibility in their interventions with patients.

## Discussion

### Overview of the findings

This study evaluates both volunteer and patient perceptions of the implementation of telephone calls, known as courtesy phone calls, by volunteers to patients in an attempt to limit the effects of the ban on family visits during the first wave of the COVID-19 pandemic.

### Strengths of the intervention

Both the patients and the volunteers greatly appreciated receiving and placing the courtesy phone calls. For the patients, what they appreciated most was being able to feel less isolated, and being able to establish a relationship, even a short one, with someone who was willing to listen and talk with them. For the volunteers, the calls also helped them feel less isolated and more useful during the pandemic when their presence on hospital premises was forbidden. These findings corroborate other studies of calls implemented during this pandemic. One involves telephone calls made by medical students to seniors. This study found that seniors’ feelings of social isolation were reduced [7]. In another study, a medical student volunteer program was established to provide remote social support to hospitalized patients in order to help combat isolation. This study mentions that many hospitalized patients felt isolated and asked to speak with the medical student again [11]. The other initiative was undertaken by staff members, who called patients daily during their hospital stay, to maintain a human connection with the patients while limiting face-to-face patient care interactions [8]. This study reports that upwards of 90% of the patients called said that they would appreciate receiving another call on the following day. Furthermore, patients said that they looked forward to these calls as a bright spot in their day. The differences between our study and the other studies in terms of the need for call-backs can be explained by population characteristics (long stays versus short stays) and by the characteristics of the callers, since the health professionals had already established a relationship of trust with the people called.

Another finding from this study, as noted by the volunteers, was their ability to make better contacts with male patients by telephone than they could in face-to-face visits. According to the female volunteers, this removes some of the discomfort men experience in opening up. The telephone medium makes them less reluctant to express themselves and talk about themselves.

Moreover, the initiative is quick and easy to deploy when organizations have a structured department of trained volunteers supported by the executive office and senior leadership. Volunteers were easily recruited in sufficient numbers, their retention rate was good over the study period, the equipment used was already in place, and the relatively short training responded to the needs in the field. The only thing added was the coordination of calls by a volunteer manager.

### Limitations of the intervention

There were a number of difficulties encountered with telephone calls made by volunteers in a short-stay hospital. The volunteers remarked that it was more difficult to get in touch with patients, to speak with them again, and to adjust their approach based on the individual’s personal situation on a local call, knowing the patient’s environment. In addition, not all patients needed courtesy calls: some were in a clinical condition that prevented them from answering the phone, and others had a social network that already served this purpose through phone calls. Some patients also underscored how it can be difficult to establish a connection with a new person on each call, or that they would have preferred calls from professionals. To our knowledge, the literature does not mention such results.

This same observation was made by the volunteers, which led them to reflect on the nature of their intervention. There was a certain tension between those who would like to establish a relationship beyond a single call and those who think that they must maintain a certain distance from the hospitalized person to avoid entering into a helping relationship. This issue was not resolved, and probably requires a nuanced response that would allow for several types of courtesy calls.

### Improving the intervention

The platform used to stay in touch with hospitalized patients was basic, and consisted of using a web platform and the telephones already in the hospital rooms, with no video. However, during the pandemic, CHUM received smartphones to encourage contact between patients and their families and between patients and healthcare professionals. They were deployed in the first wave, around the same time as the courtesy phone calls. These phones could also be used to allow for visual contact through a video connection if the person so desires. Such initiatives have been implemented in other locations where volunteers were able to use telehealth platforms to continue their activities during the COVID-19 pandemic [18]. Such virtual volunteering offers emotional support and comfort to patients who do not have friends or family they can contact or who cannot contact their loved ones due to a lack of access to technology.

### Interest in maintaining the intervention

The patients who received the calls and did not have a social network were in favour of maintaining such an initiative. As for the volunteers, although they unanimously preferred face-to-face visits, they recognized that these calls were useful in situations where their physical presence is not possible or as a complement to site visits, as they allow them to maintain their commitment and ensure that they reach as many hospitalized people as possible. Furthermore, both during and after this pandemic, virtual volunteering can protect immunocompromised, isolated, or otherwise high-risk patients and volunteers, while continuing to allow them to either receive or provide emotional and educational services.

So even if these initiatives were developed during a pandemic, earlier experiments have shown the value of being able to maintain contact with people who are isolated. For example, this is the case in mental health, where psychosocial interventions using technologies such as "Phone Pal" allow people experiencing a psychotic episode to establish a remote connection with volunteers [19] and help them feel less isolated.

### Limitations of our study

This study was carried out during the first wave of COVID-19 at CHUM. A follow-up is therefore required to ensure that we can reproduce the results at another time and will not see a decline in the effect during the second wave of the pandemic. Other limitations include the low questionnaire response rate for the patients that can be explain by the difficulties encountered reaching hospitalized patients by phone, either because of their health condition or because they have already been discharged from hospital. Although the participation rate in this study was 28.5%, this is similar to the rate in a Canadian survey conducted in healthcare facilities among adults hospitalized for maternity, medical, or acute surgical care, where the response rates for telephone conversations in Ontario and Alberta were 18% and 44%, respectively [20].

Another limitation is our inability to assess the impact of these calls on the perceived isolation of hospitalized patients before and after the call, and whether the number of calls received could affect this dimension. Finally, our focus group collected the perceptions of seven volunteers without knowing if we had reached the point of data saturation. However, the volunteer manager validated the results and did not add any other themes.

Although it is always difficult to generalize results from a single study, especially since our patient response rate is quite low, we are confident that similar results would be found in other contexts because they highlight subtleties that provide for a good understanding of the phenomenon under study.

### Recommendations

In light of our findings, we believe that these calls are relevant during a pandemic to limit the risk of infection and reduce feelings of isolation among all hospitalized patients. This measure can be enhanced through the use of smartphones, which also allow visual contact between patients and volunteers during a pandemic. Outside the pandemic context, these calls can complement volunteer site visits to reach at-risk populations, such as people who need to be in isolation. Finally, it would be worthwhile to measure the impact of these calls on anxiety through PROMs such as HADS [21] and people’s ability to regain more control over their health.

## Conclusion

Our data suggest that the courtesy calls made by the volunteers made patients feel less isolated during their hospitalization. Furthermore, these calls met the needs of patients. As for the volunteers, the intervention allowed them to maintain their commitment to patients and feel useful. These calls can also be implemented in other contexts besides a pandemic as a way for people who are isolated for medical reasons to continue having social interactions.

## Supporting information

S1 File Patients questionnaire–English version. (DOC)

S2 File Patients questionnaire–French version. (DOC)

S3 File Volunteers questionnaire–English version. (DOC)

S4 File Volunteers questionnaire–French version. (DOC)

S5 File Volunteers focus group guide. (DOC)

S1 Database Patients database. (XLSX)

S2 Database Volunteers database. (XLSX)
