# Supplementary material for: Reducing social isolation during the COVID-19 pandemic: Assessing the contribution of courtesy phone calls by volunteers
Source: PLoS One. 2022 May 4;17(5):e0266328. doi: 10.1371/journal.pone.0266328 (PMC9067884; doi:10.1371/journal.pone.0266328)
Supplement: S1 File — (DOC) [file pone.0266328.s001.DOC]

**Patient questionnaire**

The purpose of the following questions is to assess your perceptions in the context of the COVID-19 health crisis.

| 1. | The courtesy calls from volunteers (e.g., on Zoom or on the phone) meet my needs. | Completely disagree  Somewhat disagree  Neutral  Somewhat agree  Completely agree  I don’t want to answer / I don’t know / Does not apply |
| --- | --- | --- |
| 2. | What bothers you about the courtesy calls from volunteers (select all that apply)? | Nothing  Lack of interest in these calls  Fear that the information discussed would not be kept confidential  Too many calls  Not enough calls  The calls were too long  The calls were too short  Inappropriate timing of calls  A different person on each call  Difficulties understanding  Other (please specify)  I don’t want to answer / I don’t know / Does not apply |
| 3. | I believe that I created a bond of trust with the volunteer. | Completely disagree  Somewhat disagree  Neutral  Somewhat agree  Completely agree  I don’t want to answer / I don’t know / Does not apply |
| 4. | My discussions with the volunteer have made me feel less isolated. | Completely disagree  Somewhat disagree  Neutral  Somewhat agree  Completely agree  I don’t want to answer / I don’t know / Does not apply |
| 5. | Patient’s sex | Female  Male  Other |
| 6. | Patient’s age |  |
| 7. | Does the patient have or has the patient had COVID-19? | Yes  No  I don’t know / I don’t want to answer |
| 8. | Which of the following situations best describes your household? | I live alone.  I live with someone. |
|  | Do you have any comments you would like to add or share? |  |

Thank you for your cooperation.
