# Supplementary material for: Reducing social isolation during the COVID-19 pandemic: Assessing the contribution of courtesy phone calls by volunteers
Source: PLoS One. 2022 May 4;17(5):e0266328. doi: 10.1371/journal.pone.0266328 (PMC9067884; doi:10.1371/journal.pone.0266328)
Supplement: S2 File — (DOC) [file pone.0266328.s002.DOC]

**Questionnaire pour les patients**

Les questions qui suivent ont pour objectif d'évaluer votre perception dans le contexte de la crise sanitaire de la covid-19.

| 1. | Les appels de courtoisie des bénévoles (ex. sur Zoom ou au téléphone) répondent bien à mes besoins | Complètement en désaccord  Un peu en désaccord  Neutre  Un peu en accord  Complétement d’accord  Je ne souhaite pas répondre / Je ne sais pas / Ne s’applique pas |
| --- | --- | --- |
| 2. | Qu'est-ce qui vous gêne par rapport aux appels de courtoisie des bénévoles ? (Plusieurs choix de réponses possibles) | Aucun problème rencontré  Manque d’intérêt pour ces appels  Crainte du manque de confidentialité des informations échangées  Trop d’appels reçus  Pas assez d’appels reçus  Durée des appels trop longue  Durée des appels trop coute  Moment de l’appel pas approprié  Personne différente à chaque appel  Difficulté de compréhension  Autre problème, Veuillez préciser  Je ne souhaite pas répondre / Je ne sais pas / Ne s’applique pas |
| 3. | Je pense que j’ai créé un lien de confiance avec le bénévole | Complètement en désaccord  Un peu en désaccord  Neutre  Un peu en accord  Complétement d’accord  Je ne souhaite pas répondre / Je ne sais pas / Ne s’applique pas |
| 4. | Grâce à mes échanges avec le bénévole, je me sens moins isolé(e) | Complètement en désaccord  Un peu en désaccord  Neutre  Un peu en accord  Complétement d’accord  Je ne souhaite pas répondre / Je ne sais pas / Ne s’applique pas |
| 5. | Genre du patient | Féminin  Masculin  Autre |
| 6. | Âge du patient |  |
| 7. | Est-ce que le patient est ou a été atteint du COVID-19 ? | Oui  Non  Je ne sais pas / Je ne souhaite pas répondre |
| 8. | Quel type de situation correspond le mieux à la composition de votre foyer (ménage) | Je vis seul  Je vis avec quelqu’un |
|  | Avez-vous des commentaires que vous souhaitez-ajouter / partager. |  |

Merci de votre collaboration
