# Supplementary material for: Reducing social isolation during the COVID-19 pandemic: Assessing the contribution of courtesy phone calls by volunteers
Source: PLoS One. 2022 May 4;17(5):e0266328. doi: 10.1371/journal.pone.0266328 (PMC9067884; doi:10.1371/journal.pone.0266328)
Supplement: S3 File — (DOC) [file pone.0266328.s003.DOC]

Volunteers questionnaire

1. The patients-volunteers-patient partners virtual Completely disagree

community platform (e.g. on Zoom or by phone) met Somewhat disagree


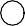

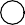

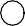

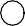

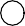

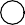


my needs as well as the needs of patients. Neutral

Somewhat agree Completely agree

I don’t want to answer/I don’t know/NA

2. The process used to connect me with patients met Completely disagree my needs. Somewhat disagree

Neutral


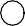

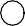

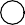

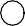

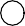

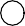


Somewhat agree Completely agree

I don’t want to answer/I don’t know/NA

3. The means used to communicate with patients (Zoom or Completely disagree phone) met my needs. Somewhat disagree

Neutral


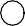

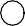

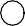

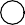

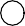

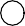


Somewhat agree Completely agree

I don’t want to answer/I don’t know/NA

4. The discussions with other volunteers (e.g. during Completely disagree webinars) met my needs. Somewhat disagree

Neutral


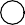

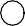

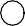

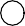

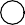

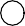


Somewhat agree Completely agree

I don’t want to answer/I don’t know/NA

5. My involvement as a volunteer has had the following Giving to others what I myself had received positive impact(s) on me (select all that apply): Making me feel useful

Making me feel valued


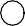

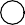

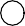

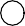

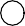

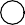

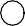


Providing relief from my isolation

Developing new skills No impact

Other

If you checked “Other,” please specify.

6. My involvement as a volunteer has had the following
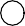
 Not feeling sufficiently supported in my negative impact(s) on me (select all that apply): intervention

Not having the necessary training Not having the information I required Not fully understanding my contribution Having too many responsibilities


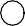

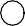

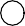

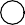

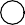

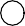

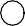

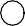


Having too many constraints

Not feeling up to the task No negative impact

Other

If you checked “Other,” please specify.

7. As a result of my interventions, I think that patients Completely disagree


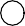

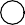

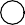

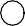

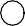

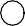


felt less isolated. Somewhat disagree Neutral

Somewhat agree Completely agree

I don’t want to answer/I don’t know/NA

8. In the context of the Covid-19 health crisis, overall, Completely disagree

I’m satisfied with my interventions. Somewhat disagree


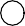

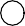

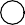

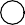

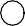

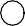


Neutral

Somewhat agree Completely agree

I don’t want to answer/I don’t know/NA

9. The training I received was sufficient for my Completely disagree

interventions with patients. Somewhat disagree Neutral


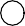

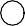

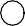

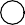

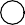

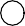


Somewhat agree Completely agree

I don’t want to answer/I don’t know/NA

10. I felt sufficiently well-equipped to meet the needs Completely disagree

of patients. Somewhat disagree Neutral


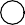

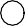

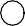

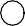

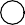

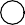


Somewhat agree Completely agree

I don’t want to answer/I don’t know/NA

11. I felt comfortable with my roles and responsibilities. Completely disagree

Somewhat disagree Neutral


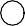

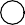

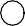

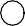

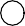

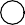


Somewhat agree Completely agree

I don’t want to answer/I don’t know/NA

12. What is your sex? Female

Male Other


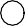

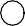

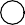


13. What is your age group? 24 years or younger 25 - 34 years

35 - 44 years


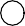

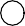

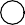

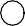

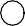

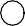

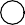

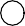


45 - 54 years

55 - 64 years

65 - 74 years

75 - 84 years 85 years or older

Do you have any comments or information you would like to share?
