# Supplementary material for: Reducing social isolation during the COVID-19 pandemic: Assessing the contribution of courtesy phone calls by volunteers
Source: PLoS One. 2022 May 4;17(5):e0266328. doi: 10.1371/journal.pone.0266328 (PMC9067884; doi:10.1371/journal.pone.0266328)
Supplement: S4 File — (DOC) [file pone.0266328.s004.DOC]

Questionnaire des bénévoles

1. La plateforme communauté virtuelle Complètement en désaccord

patients-bénévoles- patients partenaires (ex. sur Un peu en désaccord


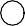

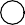

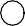

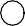

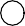

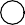


Zoom ou téléphone) ont bien répondu à mes Neutre

besoins et à ceux des patients. Un peu en accord Complètement d'accord

Je ne souhaite pas répondre/Je ne sais pas/NA

2. Le processus mis en place pour me mettre en contact Complètement en désaccord avec les patients a répondu à mes besoins Un peu en désaccord

Neutre


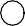

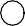

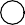

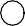

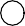

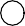


Un peu en accord Complètement d'accord

Je ne souhaite pas répondre/Je ne sais pas/NA

3. Le moyen de communication avec les patients (Zoom ou Complètement en désaccord téléphone) a répondu à mes besoins Un peu en désaccord

Neutre


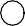

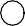

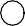

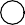

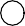

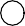


Un peu en accord Complètement d'accord

Je ne souhaite pas répondre/Je ne sais pas/NA

4. Les échanges entre bénévoles ont répondu à mes Complètement en désaccord besoins (exemple: durant les webinaires). Un peu en désaccord

Neutre


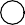

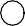

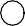

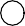

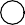

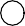


Un peu en accord Complètement d'accord

Je ne souhaite pas répondre/Je ne sais pas/NA

5. Mon implication en tant que bénévole a eu comme Rendre au prochain ce que j'ai reçu impact(s) positif(s) pour moi-même de : (plusieurs Me rendre utile

choix de réponses possible) Me valoriser


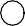

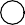

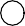

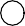

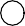

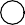

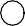


Briser mon isolement

Développer de nouvelles compétences Aucun impact positif

Autre

Si vous avez coché "Autre", veuillez préciser svp.

6. Mon implication en tant que bénévole a eu comme
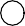
 Ne pas me sentir assez soutenu dans mon impact(s) négatif(s) pour moi-même de : (plusieurs intervention

choix de réponses possible) Ne pas avoir eu la formation nécessaire Ne pas avoir eu l'information nécessaire Ne pas bien comprendre ma contribution Avoir trop de responsabilités


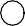

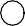

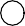

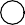

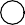

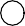

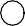

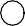


Avoir trop de contraintes

Ne pas me sentir à la hauteur de la situation Aucun impact négatif

Autre

Si vous avez coché "Autre", veuillez préciser svp.

7. Grâce à mes interventions, je pense que les Complètement en désaccord


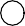

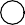

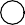

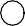

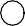

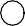


patients se sont sentis moins isolés Un peu en désaccord Neutre

Un peu en accord Complètement d'accord

Je ne souhaite pas répondre/Je ne sais pas/NA

8. Dans le contexte de la crise sanitaire de la Complètement en désaccord

Covid-19, globalement, je suis satisfait des Un peu en désaccord


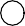

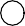

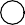

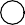

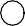

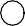


interventions que j'ai fait. Neutre

Un peu en accord Complètement d'accord

Je ne souhaite pas répondre/Je ne sais pas/NA

9. La formation que j'ai reçue était suffisante pour Complètement en désaccord

mes interventions avec les patients Un peu en désaccord Neutre


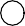

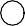

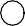

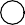

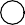

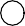


Un peu en accord Complètement d'accord

Je ne souhaite pas répondre/Je ne sais pas/NA

10. Je me suis senti(e) suffisamment outillé(e) pour Complètement en désaccord

répondre aux besoins des patients. Un peu en désaccord Neutre


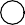

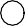

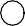

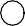

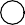

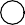


Un peu en accord Complètement d'accord

Je ne souhaite pas répondre/Je ne sais pas/NA

11. Je me suis senti(e) à l'aise avec les rôles et Complètement en désaccord

responsabilités qui m'étaient confiés. Un peu en désaccord Neutre


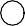

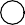

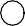

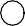

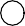

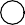


Un peu en accord Complètement d'accord

Je ne souhaite pas répondre/Je ne sais pas/NA

12. Quel est votre genre ? Féminin

Masculin Autre


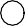

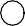

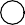


13. Quel est votre groupe d'âge ? 24 ans et moins 25 - 34 ans

35 - 44 ans


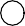

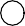

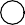

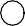

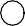

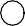

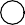

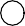


45 - 54 ans

55 - 64 ans

65 - 74 ans

75 - 84 ans 85 ans et plus

Avez-vous des commentaires ou informations que vous souhaitez partager ?
