# Supplementary material for: Reducing social isolation during the COVID-19 pandemic: Assessing the contribution of courtesy phone calls by volunteers
Source: PLoS One. 2022 May 4;17(5):e0266328. doi: 10.1371/journal.pone.0266328 (PMC9067884; doi:10.1371/journal.pone.0266328)
Supplement: S5 File — (DOC) [file pone.0266328.s005.DOC]

**Interview Guide for the Volunteer Focus Group**

**Courtesy Calls**

1. What motivated you to take part in the virtual community?
2. What did you appreciate the most about these courtesy calls?
3. What did you like the least about these courtesy calls? What challenges did you face?
4. What differences do you see between making phone calls and in-person visits?
5. Should such an intervention be continued after COVID-19? How much would you be interested in continuing to volunteer, either by phone or in person?
6. Why do you think so few people showed an interest in the virtual community, knowing that approximately 70 out of 1,000 people volunteered?
7. Among the comments received from patients, some said they would like to always have the same volunteer on the phone, to start building trust over time. How does a relationship of trust develop between you and a patient during the courtesy calls?
